# Associação Individual e Simultânea entre Fatores de Risco para Doença Cardiovascular e Hábitos Inadequados do Estilo de Vida em uma Amostra do Brasil

**DOI:** 10.36660/abc.20240149

**Published:** 2024-10-08

**Authors:** Letícia Gonçalves, Suellem Zanlorenci, Andreia Pelegrini, Tiago Rodrigues de Lima, Diego Augusto Santos Silva

**Affiliations:** 1 Universidade Federal de Santa Catarina Florianópolis SC Brasil Universidade Federal de Santa Catarina, Florianópolis, SC – Brasil; 2 Universidade do Estado de Santa Catarina Florianópolis SC Brasil Universidade do Estado de Santa Catarina, Florianópolis, SC – Brasil

**Keywords:** Saúde do Adulto, Saúde do Idoso, Comportamentos de Risco à Saúde, Fatores de Risco de Doenças Cardíacas

## Abstract

**Fundamento:**

As doenças cardiovasculares (DCV) são frequentemente influenciadas por fatores modificáveis, principalmente pelas escolhas de estilo de vida dos indivíduos, que desempenham um papel crucial na modulação do risco cardiovascular.

**Objetivo:**

Investigar a associação individual e simultânea entre comportamentos inadequados do estilo de vida e fatores de risco para DCV em adultos e idosos.

**Métodos:**

Trata-se de um estudo transversal com 1.079 usuários do Programa Academia da Saúde no Brasil. Foram investigadas individual e coletivamente informações relacionadas a dieta inadequada, consumo excessivo de álcool, tabagismo e inatividade física (0, 1 ou ≥ 2 fatores) em associação com fatores de risco para DCV (hipertensão, hipercolesterolemia, diabetes mellitus e obesidade), considerando os dois desfechos seguintes: presença de fatores de risco para DCV e número de fatores de risco para DCV presentes no mesmo indivíduo (0, 1, 2 ou ≥ 3 fatores de risco). Foram utilizadas análises de regressão logística e multinomial. A significância estatística adotada foi de 5%.

**Resultados:**

Um maior número de comportamentos do estilo de vida inadequados foi associado a maiores chances de presença simultânea de 1, 2 ou ≥ 3 fatores de risco de DCV. A adoção simultânea de 1 e ≥ 2 comportamentos de estilo de vida inadequados foi associada a maiores chances de hipercolesterolemia. A adesão simultânea a ≥ 2 comportamentos de estilo de vida inadequados foi associada a menores chances de hipertensão.

**Conclusão:**

Um maior número de comportamentos de estilo de vida inadequados foi associado a maiores chances de presença simultânea de múltiplos fatores de risco de DCV.

## Graphical abstract

**Figure:**
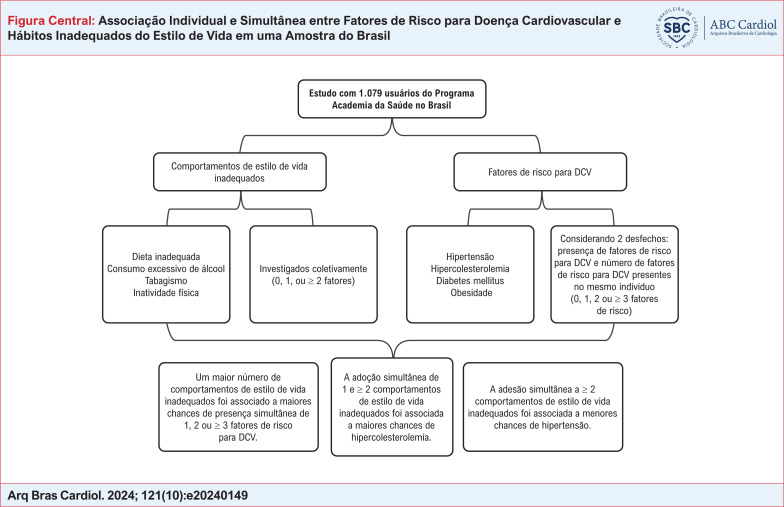
Associação Individual e Simultânea entre Fatores de Risco para Doença Cardiovascular e Hábitos Inadequados do Estilo de Vida em uma Amostra do Brasil. DCV: doenças cardiovasculares.

## Introdução

As doenças cardiovasculares (DCV) são uma preocupação constante para a saúde pública e estão entre as principais causas de morbimortalidade em todo o mundo.^1,2^ Essas condições são frequentemente influenciadas por fatores modificáveis, principalmente as escolhas de estilo de vida dos indivíduos, que desempenham um papel crucial na modulação do risco cardiovascular.^3,4^ Tabagismo,^5^ consumo excessivo de álcool,^6^ inatividade física^3^ e hábitos alimentares inadequados^7^ têm sido identificados como os principais elementos responsáveis pela maioria das mortes relacionadas a doenças não transmissíveis.^2,8^

Embora a literatura revele uma associação entre consumo de álcool e DCV, essa relação ainda é considerada complexa e, às vezes, paradoxal.^6^ Por um lado, o consumo modesto de bebidas alcoólicas tem sido associado a menor incidência e mortalidade por DCV; por outro lado, o consumo excessivo de bebidas alcoólicas aumenta substancialmente o risco de DCV.^9^

O tabagismo é outra variável que tem sido associada a um risco aumentado de desfechos diretamente relacionados às DCV,^10,11^ incluindo acidente vascular cerebral, infarto agudo do miocárdio, doença cardíaca coronária, doença arterial periférica, insuficiência cardíaca e hipertensão.^11^ O tabagismo também é uma das principais causas do estado inflamatório crônico no corpo, que contribui para o desenvolvimento de processos de doenças aterogênicas e aumenta os níveis de biomarcadores inflamatórios, conhecidos como indicadores de eventos cardiovasculares.^10^

A inatividade física tem sido considerada um fator de risco bem estabelecido para o desenvolvimento de DCV, conforme destacado pelo Departamento de Saúde e Serviços Humanos dos Estados Unidos em 2018.^12^ A atividade física regular não só tem um impacto positivo nos níveis de pressão arterial,^13^ mas também desempenha um papel crucial na redução do risco de obesidade e diabetes.^14^

Os padrões alimentares têm um impacto significativo em vários fatores de risco relacionados à saúde cardiometabólica, abrangendo condições como doenças cardíacas, acidente vascular cerebral e diabetes tipo 2.^15^ Essas doenças coletivamente geram impactos consideráveis tanto na saúde pública quanto na economia.^16^ Entender como os elementos alimentares se relacionam com as doenças cardiometabólicas é fundamental para estabelecer prioridades, direcionar políticas de saúde pública e desenvolver estratégias que promovam mudanças nos hábitos alimentares, visando melhorar a saúde.

Considerando que hábitos de vida pouco saudáveis podem ser modificados^17^ e que os indivíduos tendem a exibir simultaneamente mais de um hábito de vida pouco saudável,^18^ a investigação de tais interrelações contribui para a identificação de subgrupos de indivíduos mais propensos às DCV. Isso é particularmente importante, pois um maior número de hábitos de vida não saudáveis está diretamente relacionado a um risco aumentado de DCV.^11,19^

Portanto, o objetivo do presente estudo foi investigar a associação individual e simultânea entre comportamentos de estilo de vida inadequados (dieta inadequada, consumo excessivo de álcool, tabagismo e inatividade física) com fatores de risco para DCV (hipertensão, hipercolesterolemia, diabetes mellitus e obesidade) em uma amostra de adultos e idosos do Brasil.

## Métodos

### Desenho do estudo, considerações éticas e participantes

O presente estudo transversal utilizou dados do estudo populacional denominado "MOTIVA-SUS: estudo epidemiológico transversal sobre os determinantes motivacionais para a prática de atividade física em usuários do Programa Academia da Saúde." O estudo foi aprovado pelo Comitê de Ética em Pesquisa em Seres Humanos da Universidade Federal de Santa Catarina. A coleta de dados foi realizada entre fevereiro e agosto de 2022, por meio de entrevistas telefônicas, devido à situação da pandemia de COVID-19. Todos os participantes da pesquisa receberam informações contidas no termo de consentimento livre e esclarecido e somente aqueles que concordaram com as informações participaram da entrevista. O estudo incluiu uma amostra representativa de indivíduos com 18 anos ou mais, de ambos os sexos, usuários do Programa Academia da Saúde das cinco regiões geográficas do território brasileiro (Centro-Oeste, Nordeste, Norte, Sudeste e Sul). A descrição do processo de amostragem é detalhada no Material Suplementar 1.

Foram considerados os seguintes critérios de exclusão: indivíduos acamados, amputados, engessados e indivíduos que não tinham capacidade cognitiva para se comunicar por contato telefônico. Recusas ou perdas foram consideradas para indivíduos elegíveis que não puderam ser contactados após um mínimo de quatro chamadas, uma das quais foi no fim de semana e outra à noite. Adicionalmente, recusas foram consideradas quando indivíduos elegíveis optaram por não participar do estudo, mesmo após esclarecimento sobre o propósito da pesquisa.

### Fatores de risco para doença cardiovascular

O índice de massa corporal (IMC) foi inicialmente observado como uma variável contínua e subsequentemente dicotomizado. Os indivíduos autorrelataram massa corporal e altura. A partir dessas informações, foi calculado o IMC (kg/m^2^). A obesidade foi classificada como IMC ≥ 30,0 kg/m^2^.^20^

As informações obtidas nas entrevistas foram utilizadas para identificar indivíduos com hipertensão, dislipidemia e diabetes mellitus, com base em respostas afirmativas à seguinte pergunta sobre diagnóstico médico autorrelatado: "Algum médico ou profissional de saúde já lhe disse que o(a) Sr.(a) tem…?". Adicionalmente, o uso de medicação específica para cada uma dessas condições foi utilizado para classificar os indivíduos como positivos para os fatores de risco investigados.

### Comportamentos de estilo de vida inadequados

As variáveis relacionadas à adoção de comportamentos de estilo de vida inadequados (tabagismo, consumo excessivo de álcool, inatividade física e dieta inadequada) foram coletadas com base em perguntas derivadas do questionário da Vigilância de Fatores de Risco e Proteção para Doenças Crônicas por Inquérito Telefônico (VIGITEL).^21^

O tabagismo foi avaliado por meio da seguinte pergunta: "Atualmente, o(a) Sr.(a) fuma?", com opções de resposta "não" ou "sim". Indivíduos que responderam "sim" foram considerados fumantes, independentemente da intensidade ou frequência do hábito de fumar.^21^

O consumo excessivo de álcool foi definido como a ingestão de 4 ou mais doses de bebida alcoólica em uma única ocasião, pelo menos uma vez nos últimos 30 dias, avaliado pela resposta afirmativa à seguinte pergunta: "Nos últimos 30 dias, o(a) Sr.(a) chegou a consumir 4 ou mais doses de bebida alcoólica em uma única ocasião?". Embora a literatura não apresente um consenso definido sobre as recomendações de consumo de álcool para adultos,^20^ uma métrica frequentemente adotada para caracterizar o consumo excessivo é a ingestão de 4 ou mais doses de bebida alcoólica em uma única ocasião, ocorrida pelo menos uma vez nos últimos 30 dias.^20^

A inatividade física foi avaliada com base no domínio lazer, utilizando a seguinte pergunta: "Nos últimos 3 meses, o(a) Sr.(a) praticou algum tipo de exercício físico ou esporte?". As opções de resposta eram "não" ou "sim". Segundo recomendações da literatura, os indivíduos foram classificados como "fisicamente inativos" se não tivessem praticado ou realizado nenhuma atividade física no lazer nos últimos 3 meses.^21,22^ A reprodutibilidade das perguntas da VIGITEL relacionadas à atividade física apresentou especificidade de 72,0% a 91,2% e sensibilidade de 54,8% a 67,7% para indivíduos inativos, demonstrando confiabilidade nesse domínio.^22^

As variáveis associadas aos hábitos alimentares abrangeram a frequência de consumo alimentar no dia anterior à entrevista, que se limitaram, para fins do presente estudo, aos grupos de alimentos industrializados/ultraprocessados,^21,23^ obtidos a partir das seguintes perguntas: "Agora vou listar alguns alimentos e gostaria que o(a) Sr.(a) me dissesse se comeu algum deles ontem (desde quando acordou até quando foi dormir): refrigerante; suco de fruta em caixa, caixinha ou lata; refresco em pó; bebida achocolatada; iogurte com sabor; salgadinho de pacote (ou *chips*) ou biscoito/bolacha salgado; biscoitos/bolacha doce, biscoito recheado ou bolinho de pacote; chocolate, sorvete, gelatina ou outra sobremesa industrializada; salsicha, linguiça, mortadela ou presunto; pão de forma, pão de cachorro-quente ou pão de hambúrguer; maionese, ketchup ou mostarda; margarina; macarrão instantâneo, sopa de pacote, lasanha congelada ou outro prato pronto comprado congelado." As opções de resposta eram "sim" ou "não". Aqueles que consumiram 5 ou mais grupos de alimentos ultraprocessados no dia anterior à entrevista foram considerados como tendo uma "dieta inadequada".^21^

As informações sobre comportamentos de estilo de vida inadequados foram investigadas tanto individual quanto coletivamente (de acordo com o número de comportamentos inadequados). Para esse propósito, o número de comportamentos de estilo de vida inadequados adotados por cada participante (dieta inadequada, consumo excessivo de álcool, tabagismo e inatividade física) foi somado e transformado em uma escala ordinal (0 = nenhum comportamento de estilo de vida inadequado; 1 = 1 comportamento de estilo de vida inadequado; 2 = 2 comportamentos de estilo de vida inadequados; 3 = 3 comportamentos de estilo de vida inadequados). No entanto, como um pequeno número de indivíduos adotou 3 comportamentos de estilo de vida inadequados simultaneamente (n = 12; 1,1%), para fins de análise, as categorias 2 e 3 foram agrupadas (0, 1 ou ≥ 2 comportamentos de estilo de vida inadequados).

### Variáveis dependentes

Dois desfechos diferentes foram considerados para o presente estudo: fatores de risco para DCV (hipertensão, dislipidemia, diabetes mellitus e obesidade) e o número de fatores de risco para DCV presentes no mesmo indivíduo (0, 1, 2, 3 ou 4 fatores de risco). Como menos de 3,0% (n = 31) da amostra tinham 4 fatores de risco para DCV, eles foram agrupados na categoria de 3 fatores de risco para DCV. Dessa maneira, para fins de análise, os participantes deste estudo foram classificados como tendo 0, 1, 2 ou ≥ 3 fatores de risco para DCV.

### Variáveis sociodemográficas e de saúde cardiovascular

As variáveis de controle analisadas incluíram sexo (masculino/feminino), faixa etária (adultos, definidos como 20 a 59 anos de idade; idosos, definidos como 60 anos ou mais), escolaridade (0 a 8 anos, 9 a 11 anos, 12 anos ou mais) e estado civil (solteiro, casado/união estável, separado/divorciado/viúvo). Com base no diagnóstico médico autorrelatado, adultos e idosos foram classificados como positivos para DCV quando afirmaram ter histórico de acidente vascular cerebral, isquemia cerebral ou doença cardíaca.

### Análise de dados

Estatísticas descritivas foram usadas para descrever as informações analisadas. Variáveis categóricas são descritas por meio de frequências absolutas e relativas. O teste qui-quadrado de heterogeneidade foi usado para identificar possíveis diferenças entre os grupos de acordo com a presença individual e o número de fatores de risco para DCV presentes no mesmo indivíduo.

A regressão logística binária foi usada para testar a associação entre a presença de fatores de risco para DCV (hipertensão, hipercolesterolemia, diabetes mellitus e obesidade) como variáveis dependentes com a adoção individual e simultânea (0, 1, ≥ 2 hábitos) de hábitos de vida inadequados (tabagismo, inatividade física, dieta inadequada e consumo excessivo de álcool) pelo mesmo indivíduo como variáveis independentes. Para esse modelo de regressão, os resultados foram expressos como *odds ratio* (OR) com intervalo de confiança (IC) de 95%, com a categoria de referência sendo ausência da doença.

Adicionalmente, a análise de regressão logística multinomial foi empregada para investigar a associação entre a presença simultânea de fatores de risco para DCV no mesmo indivíduo (variável dependente) com adoção individual e adoção simultânea de comportamentos de estilo de vida inadequados (variáveis independentes), com resultados expressos como OR e IC de 95%, com a categoria de referência sendo 0 fatores de risco de DCV.

As análises de associação foram ajustadas considerando sexo, faixa etária, anos de escolaridade, estado civil e DCV (doença cardiovascular, isquemia cerebral e acidente vascular cerebral), independentemente do nível de significância estatística da análise bruta em associação com os desfechos.

As análises foram realizadas considerando os pesos amostrais e o desenho do estudo. A análise dos dados foi realizada usando o software Stata (Stata Corp LP, College Station, Texas, EUA), versão 16.0, com um valor de p < 0,05 sendo considerado estatisticamente significativo.

## Resultados

O estudo incluiu 1.079 indivíduos com informações completas para todas as variáveis investigadas (89,0% da amostra original). Do total avaliado, 40,4% eram hipertensos, 36,6% tinham hipercolesterolemia, 20,0% tinham diabetes mellitus e 32,9% eram obesos. Além disso, 28,4% da amostra não apresentava nenhum fator de risco para DCV, enquanto 31,4% tinham 1 fator de risco para DCV, 24,9% tinham 2 fatores de risco para DCV e 15,3% tinham 3 ou mais fatores de risco para DCV simultaneamente (Tabela 1). A Tabela 2 apresenta a distribuição da amostra para cada um dos fatores de risco para DCV investigados.

**Tabela 1:** Informações descritivas sobre os participantes do Programa Academia da Saúde. Brasil, 2022

Variáveis		n	% (IC 95%)
**Sexo**			
	Masculino	81	6,8 (2,8-15,7)
	Feminino	998	93,2 (84,3-97,2)
**Idade (anos)**			
	20-59	804	74,4 (55,1-87,3)
	≥ 60	275	25,6 (12,6-44,9)
**Anos de estudo**			
	0-8	204	18,9 (11,7-28,4)
	9-11	216	20,0 (18,9-22,4)
	12 anos ou mais	659	91,1 (50,7-69,8)
**Estado civil**			
	Solteiro	223	20,1 (10,6-34,5)
	Casado	668	62,9 (59,3-66,4)
	Separado/viúvo	188	17,0 (9,7-28,0)
**Tabagismo**			
	Não	1052	97,6 (97,1-97,9)
	Sim	27	2,4 (2,0-2,8)
**Dieta inadequada**			
	Não	931	85,3 (80,1-89,3)
	Sim	148	14,6 (10,6-19,8)
**Inatividade física**			
	Não	750	69,9 (57,8-79,7)
	Sim	329	30,1 (20,3-42,2)
**Consumo excessivo de álcool**			
	Não	908	84,3 (82,2-86,2)
	Sim	171	15,6 (13,7-17,7)
**Adoção simultânea de hábitos de vida não saudáveis**			
	0	327	29,5 (24,3-35,2)
	1	634	59,3 (57,5-61,0)
	≥ 2	118	11,2 (7,9-15,6)
**Hipertensão arterial**			
	Não	655	59,6 (49,6-68,8)
	Sim	424	40,4 (31,2-50,4)
**Hipercolesterolemia**			
	Não	689	63,4 (57,5-69,9)
	Sim	390	36,6 (31,1-42,5)
**Diabetes mellitus**			
	Não	877	80,0 (76,0-83,4)
	Sim	202	20,0 (16,6-24,0)
Obesidade*			
	Não	735	67,1 (58,5-74,6)
	Sim	344	32,9 (25,4-41,4)
**Presença simultânea de fatores de risco para DCV**			
	0	324	28,4 (23,3-34,1)
	1	340	31,4 (30,2-32,6)
	2	256	24,9 (18,7-32,3)
	≥ 3	159	15,3 (14,0-16,6)
**Presença de DCV**			
	Não	987	91,8 (83,7-96,0)
	Sim	92	8,2 (3,9-16,2)

* informações geradas a partir do índice de massa corporal (≥ 30 kg/m^2^).

IC: intervalo de confiança; n: número de frequências; presença de DCV: doença cardiovascular, acidente vascular cerebral ou isquemia cerebral.

**Tabela 2:** Distribuição e frequência de hábitos de vida inadequados analisados individualmente ou como um grupo de fatores, n = 1.079

Hábitos de vida inadequados	n	(%)
Nenhum	534	49,5
Tabagismo	9	0,8
Dieta inadequada	73	6,8
Inatividade física	234	21,6
Consumo excessivo de álcool	111	10,3
Tabagismo + Dieta inadequada	3	0,3
Tabagismo + Inatividade física	8	0,7
Tabagismo + Consumo excessivo de álcool	2	0,2
Dieta inadequada + Inatividade física	45	4,2
Dieta inadequada + Consumo excessivo de álcool	18	1,7
Inatividade física + Consumo excessivo de álcool	30	2,8
Tabagismo + Dieta inadequada + Inatividade física	2	0,2
Tabagismo + Dieta inadequada + Consumo excessivo de álcool	-	-
Tabagismo + Inatividade física + Consumo excessivo de álcool	3	0,3
Dieta inadequada + Inatividade física + Consumo excessivo de álcool	7	0,6
Tabagismo + Dieta inadequada + Inatividade física + Consumo excessivo de álcool	-	-

A presença de 3 ou mais fatores de risco para DCV foi maior entre participantes do sexo feminino, com idade entre 20 e 59 anos, com 12 ou mais anos de estudo, casados, que adotaram um comportamento de estilo de vida inadequado e sem a presença de DCV (Tabela 3 e Tabela 4).

**Tabela 3:** Informações descritivas sobre características individuais e hábitos de vida segundo fatores de risco para doenças cardiovasculares em participantes do Programa Academia da Saúde. Brasil, 2022

Variáveis		Hipertensão		valor p	Hipercolesterolemia		valor p	Diabetes mellitus		valor p	Obesidade*		valor p
		n	% (IC 95%)		n	% (IC 95%)		n	% (IC 95%)		n	% (IC 95%)	
**Sexo**				<0,01†			0,99			0,51			0,13
	Masculino	35	8,4 (3,9-17,2)		27	6,8 (2,9-15,1)		18	8,9 (3,0-23,4)		21	5,6 (1,7-16,7)	
	Feminino	389	91,6 (82,8-96,1)		363	93,2 (84,9-97,1)		184	91,1 (76,6-97,0)		323	94,4 (83,3-98,3)	
**Idade (anos)**				<0,01†			<0,01†			<0,01†			<0,01†
	20-59	254	60,5 (45,1-74,1)		259	67,1 (50,0-80,5)		118	59,5 (41,6-75,2)		280	84,1 (61,8-94,5)	
	≥ 60	170	39,5 (25,8-54,9)		131	32,9 (19,5-49,9)		84	40,5 (24,7-58,4)		64	15,9 (5,5-38,2)	
**Anos de estudo**			<0,01†			0,08			<0,01†			0,84
	0-8	112	26,9 (18,4-37,6)		89	22,9 (14,5-34,2)		47	22,9 (16,7-30,6)		69	19,3 (10,5-32,6)	
	9-11	101	24,3 (20,9-27,9)		88	22,2 (17,5-27,7)		57	28,4 (26,4-30,4)		65	20,5 (16,9-24,6)	
	≥ 12	211	48,8 (38,3-59,3)		213	54,9 (49,9-59,7)		98	48,7 (41,3-56,2)		210	60,2 (51,7-68,1)	
**Estado civil**				<0,01†			<0,01†			0,13			<0,01†
	Solteiro	66	15,8 (9,3-25,6)		55	13,7 (6,7-25,8)		34	18,7 (9,8-32,7)		76	23,5 (14,1-36,4)	
	Casado	266	63,9 (62,0-65,7)		255	66,0 (65,2-66,8)		129	65,0 (62,3-67,6)		216	63,1 (60,9-65,2)	
	Separado/viúvo	92	20,2 (13,1-29,9)		80	20,3 (12,3-31,7)		39	16,3 (8,6-28,7)		52	13,4 (6,4-25,9)	
**Tabagismo**				0,21			0,06			0,15			0,96
	Não	407	96,1 (92,1-98,1)		373	95,9 (93,3-97,4)		193	95,5 (90,1-98,0)		334	97,6 (96,7-98,3)	
	Sim	17	3,9 (1,9-7,9)		17	4,1 (2,5-6,6)		9	4,5 (2,0-9,8)		10	2,4 (1,7-3,3)	
**Dieta inadequada**				0,11			0,06			0,29			0,60
	Não	373	88,4 (78,0-94,2)		335	84,5 (78,8-88,9)		176	88,0 (76,3-94,3)		294	84,4 (74,3-91,0)	
	Sim	51	11,6 (5,8-21,9)		55	15,5 (11,0-21,2)		26	12,0 (5,7-23,6)		50	15,6 (8,9-25,6)	
**Inatividade física**				0,79			0,40			0,06			0,96
	Não	298	70,2 (56,5-81,1)		278	71,7 (58,3-82,2)		151	76,4 (58,5-88,2)		241	69,9 (60,2-78,1)	
	Sim	126	29,8 (18,9-43,5)		112	28,3 (17,8-41,7)		51	23,6 (11,8-41,4)		103	30,1 (21,9-39,8)	
**Consumo excessivo de álcool**				0,88			0,03†			0,27			0,63
	Não	335	84,2 (79,5-87,9)		322	82,2 (78,7-85,1)		175	86,2 (81,3-89,9)		289	83,4 (81,2-85,4)	
	Sim	69	15,8 (12,1-20,5)		68	17,8 (14,8-21,2)		27	13,8 (10,0-18,7)		55	16,6 (14,6-18,8)	
**Adoção simultânea de hábitos de estilo de vida não saudáveis**				<0,01†			<0,01†			0,02†			0,01†
	0	0	-		91	23,8 (17,5-31,5)		43	23,2 (16,4-31,8)		87	23,7 (16,7-32,3)	
	1	384	90,7 (82,0-95,5)		251	63,4 (59,7-66,9)		138	66,5 (60,6-71,9)		218	64,5 (59,5-69,2)	
	≥ 2	40	9,3 (4,5-18,0)		48	12,8 (9,3-17,3)		21	10,3 (6,4-16,2)		39	11,8 (8,9-15,4)	
**Presença de DCV**				<0,01†			0,09			0,02†			0,31
	Não	363	86,1 (74,6-92,9)		345	89,5 (77,3-95,5)		174	86,9 (73,5-94,1)		309	90,4 (76,7-96,3)	
	Sim	61	13,9 (7,1-25,3)		45	10,5 (4,4-22,6)		28	13,1 (5,9-26,4)		35	9,6 (3,6-23,2)	

Teste qui-quadrado de heterogeneidade. IC: intervalo de confiança; n: número de frequências; presença de DCV: doença cardiovascular, acidente vascular cerebral e isquemia cerebral.

* informações geradas a partir do índice de massa corporal (obesidade ≥ 30 kg/m^2^);

† p < 0,05.

**Tabela 4:** Informações descritivas sobre características individuais e hábitos de vida segundo a presença simultânea de fatores de risco para doenças cardiovasculares em participantes do Programa Academia da Saúde. Brasil, 2022

Variáveis		Presença simultânea de fatores de risco para DCV								Valor p
		0		1		2		≥ 3		
		n	% (IC 95%)	n	% (IC 95%)	n	% (IC 95%)	n	% (IC 95%)	
**Sexo**										0,04†
	Masculino	32	8,5 (2,9-21,8)	17	4,0 (1,8-8,5)	16	6,5 (2,0-18,7)	16	10,3 (3,7-25,2)	
	Feminino	292	91,5 (78,2-97,0)	323	96,0 (91,4-98,2)	240	93,5 (81,3-97,9)	143	89,7 (74,8-96,5)	
**Idade (anos)**										<0,01†
	20-59	279	86,6 (72,6-94,0)	256	73,9 (47,5-89,9)	169	65,5 (51,0-77,6)	100	67,4 (42,6-85,2)	
	≥ 60	45	13,4 (5,9-27,4)	54	26,1 (10,1-52,5)	87	34,5 (22,4-49,0)	59	32,5 (14,8-57,3)	
**Anos de estudo**										<0,01†
	0-8	44	13,4 (8,3-20,8)	59	16,0 (10,0-24,5)	55	21,9 (13,2-34,0)	46	28,8 (19,0-41,0)	
	9-11	44	13,6 (12,0-15,4)	77	23,7 (19,7-28,1)	56	22,3 (19,9-24,8)	39	24,7 (18,6-31,9)	
	≥ 12	236	72,9 (65,2-79,5)	204	60,3 (49,0-70,6)	145	55,8 (44,5-66,5)	74	46,5 (39,9-53,2)	
**Estado civil**										0,03†
	Solteiro	93	27,4 (14,9-44,9)	60	16,7 (9,3-28,2)	44	16,2 (5,9-36,9)	26	19,4 (12,8-28,3	
	Casado	186	58,3 (47,3-68,5)	223	66,2 (62,7-69,5)	153	62,0 (54,4-69,0)	106	66,3 (64,8-67,8)	
	Separado/viúvo	45	14,2 (8,9-22,0)	57	17,1 (8,9-29,9)	59	21,8 (12,5-35,2)	27	14,2 (7,9-24,3)	
**Tabagismo**										0,14
	Não	320	99,1 (94,7-99,8)	333	97,7 (93,3-99,3)	251	98,1 (93,9-99,4)	148	93,7 (87,6-96,9)	
	Sim	4	0,9 (0,1-5,3)	7	2,3 (0,7-6,7)	5	1,9 (1,0-6,1)	11	6,3 (3,1-12,3)	
**Dieta inadequada**										0,38
	Não	276	83,8 (80,7-86,4)	293	84,4 (80,0-87,9)	227	88,2 (79,7-93,4)	135	85,5 (70,0-93,7)	
	Sim	48	16,2 (13,5-19,3)	47	15,6 (12,0-19,9)	29	11,8 (6,6-20,3)	24	14,5 (6,3-29,9)	
**Inatividade física**										0,23
	Não	224	69,5 (56,9-79,7)	228	67,5 (56,4-76,9)	181	70,5 (53,2-83,4)	117	74,5 (63,0-83,3)	
	Sim	100	30,5 (20,3-43,1)	112	32,5 (23,1-43,6)	75	29,5 (16,5-46,8)	42	25,5 (16,6-37,0)	
**Consumo excessivo de álcool**										0,24
	Não	265	83,2 (73,9-89,6)	303	88,5 (82,9-92,4)	209	81,3 (79,9-82,5)	131	83,0 (75,3-88,6)	
	Sim	59	16,8 (10,3-26,0)	37	11,5 (7,6-17,1)	47	18,7 (17,5-20,1)	28	17,0 (11,3-24,7)	
**Adoção simultânea de hábitos de estilo de vida não saudáveis**										<0,01†
	0	154	47,2 (40,6-53,9)	131	37,3 (30,4-44,7)	36	14,4 (7,4-26,2)	6	4,9 (1,0-20,4)	
	1	132	42,0 (35,8-48,3)	176	51,5 (47,6-55,4)	193	73,3 (69,0-77,3)	133	84,7 (80,7-87,9)	
	≥ 2	38	10,8 (7,9-14,6)	33	11,2 (8,0-15,4)	27	12,2 (7,3-19,6)	20	10,3 (5,7-18,1)	
**Presença de DCV**										<0,01†
	Não	307	94,5 (88,2-97,5)	324	96,1 (92,6-98,0)	224	88,8 (81,7-93,4)	132	82,9 (59,5-94,1)	
	Sim	17	5,5 (2,5-11,8)	16	3,9 (1,9-7,4)	32	11,2 (6,6-18,3)	27	17,1 (5,8-40,5)	

Teste qui-quadrado de heterogeneidade; n: número de frequências; %: porcentagem; ≥: maior ou igual; Presença de DCV: doença cardiovascular, acidente vascular cerebral, acidente vascular cerebral ou isquemia cerebral; IC95%: intervalo de confiança;

† p<0,05.

Tabagismo, consumo excessivo de álcool e adoção de 1 e ≥ 2 comportamentos de estilo de vida inadequados foram associados a maiores chances de hipercolesterolemia. A adoção de 1 comportamento de estilo de vida inadequado foi associada a maiores chances de obesidade. A adoção simultânea de ≥ 2 comportamentos de estilo de vida inadequados foi associada a menores chances de hipertensão (Tabela 5).

**Tabela 5:** Análise de regressão logística binária ajustadaa entre a adoção de hábitos de vida inadequados e a presença de fatores de risco para doenças cardiovasculares em adultos e idosos usuários do Programa Academia da Saúde. Brasil, 2022

Variáveis		Hipertensão^b^		Hipercolesterolemia^b^		Diabetes mellitus^b^		Obesidade* b	
		OR	% (IC 95%)	OR	% (IC 95%)	OR	% (IC 95%)	OR	% (IC 95%)
**Tabagismo**^a^									
	Não	1		1		1		1	
	Sim	1,1	0,2-4,9	2,0	1,1-3,6†	2,3	0,9-5,8	0,7	0,4-1,5
**Inatividade física**^a^									
	Não	1		1		1		1	
	Sim	1,1	0,8-1,4	0,9	0,6-1,3	0,7	0,4-1,1	0,9	0,7-1,2
**Dieta inadequada**^a^									
	Não	1		1		1		1	
	Sim	0,6	0,4-1,2	1,1	1,0-1,3	0,7	0,4-1,3	1,1	0,7-1,8
**Consumo excessivo de álcool**^a^									
	Não	1		1		1		1	
	Sim	1,2	0,9-1,4	1,4	1,2-1,6†	0,9	0,6-1,3	1,0	0,7-1,7
**Adoção simultânea de hábitos de vida não saudáveis**^a^									
	0	‡		1		1		1	
	1	1		1,4	1,1-1,9†	1,3	0,9-1,9	1,7	1,2-2,4†
	≥2	0,3	0,2-0,6†	1,7	1,4-2,1†	1,1	0,9-1,4	1,5	0,9-2,6

IC: intervalo de confiança; OR: *odds ratio*.

* informações geradas a partir do índice de massa corporal (obesidade ≥ 30 kg/m^2^);

† p < 0,05;

‡ ausência de indivíduos que simultaneamente apresentavam hipertensão e não adotavam hábitos de vida inadequados. Para essa situação, foi utilizada como referência a categoria de indivíduos que adotaram 1 hábito de vida inadequado.

a Resultados ajustados para sexo, idade, escolaridade, estado civil e doença cardiovascular;

b Ausência da doença foi considerada como referência para as análises.

Maiores chances para a presença de 1 fator de risco de DCV foram observadas entre aqueles que adotaram 1 e ≥ 2 comportamentos de estilo de vida inadequados. Em relação aos achados relacionados à presença de 2 fatores de risco de DCV, maiores chances para essa condição foram observadas entre aqueles que tiveram 1 e ≥ 2 comportamentos de estilo de vida inadequados. Maiores chances para a presença de 3 fatores de risco de DCV foram observadas entre aqueles que adotaram 1 e ≥ 2 comportamentos de estilo de vida inadequados (Tabela 6).

**Tabela 6:** Análise de regressão logística multinominal ajustadaa entre a adoção de hábitos de vida inadequados e a presença de fatores de risco para doenças cardiovasculares em adultos e idosos usuários do Programa Academia da Saúde. Brasil, 2022

Variáveis		Adoção simultânea de hábitos de vida não saudáveis					
		1^b^		2^b^		≥ 3^b^	
		OR	% (IC 95%)	OR	% (IC 95%)	OR	% (IC 95%)
**Tabagismo**^a^							
	Não	1		1		1	
	Sim	2,1	0,6-7,1	1,7	0,3-11,9	5,8	0,5-59,4
**Inatividade física**^a^							
	Não	1		1		1	
	Sim	1,2	0,9-1,5	1,0	0,7-1,6	0,8	0,5-1,3
**Dieta inadequada**^a^							
	Não	1		1		1	
	Sim	1,0	0,8-1,3	0,7	0,4-1,2	0,9	0,4-2,1
**Consumo excessivo de álcool**^a^							
	Não	1		1		1	
	Sim	0,7	0,3-1,8	1,3	0,7-2,3	1,2	1,0-1,4
**Adoção simultânea de hábitos de vida não saudáveis**^a^							
	0	1		1		1	
	1	1,5	1,1-2,0†	5,3	3,3-8,5†	18,0	3,4-94,6†
	≥ 2	1,4	1,1-1,9†	4,0	1,9-8,3†	9,6	1,5-62,6†

DCV: doença cardiovascular; IC: intervalo de confiança; OR: odds ratio.

† p < 0,05;

a Resultados ajustados para sexo, idade, escolaridade, estado civil e doença cardiovascular;

b Presença de 0 fatores de risco para DCV foi considerada como referência para as análises.

## Discussão

O achado principal do presente estudo foi que um maior número de comportamentos de estilo de vida inadequados foi associado a maiores chances de presença simultânea de 1, 2 ou ≥ 3 fatores de risco de DCV. Os mecanismos potenciais relacionados aos efeitos nocivos à saúde cardiovascular derivados da adoção de comportamentos de estilo de vida inadequados podem estar relacionados à combinação de danos à saúde atribuídos a cada fator. A inatividade física contribui diretamente para o balanço energético positivo, que está associado à piora dos perfis lipídicos e lipoproteicos^3,24,25^ e ao aumento da adiposidade corporal,^26^ bem como à sensibilidade à insulina e aos desequilíbrios nos níveis pressóricos.^27^ O consumo excessivo de álcool pode contribuir para um aumento da pressão arterial sistólica por meio do aumento da rigidez arterial^28^ e contribuir para alterações metabólicas que podem, por sua vez, aumentar diretamente o risco de DCV.^29^ Os efeitos deletérios do tabagismo na saúde cardiovascular podem estar relacionados aos componentes químicos presentes no tabaco, como nicotina, alcatrão e monóxido de carbono.^30^ Esses componentes, além de estarem diretamente associados à ativação nervosa simpática e ao aumento da pressão arterial,^31^ intensificam a resistência à insulina, estabelecendo uma relação com o acúmulo central de gordura, o que contribui para o desenvolvimento de distúrbios metabólicos como síndrome metabólica e diabetes mellitus.^30^ Ademais, a adoção de uma dieta inadequada^32^ pode levar a desequilíbrios no metabolismo glicêmico, a um aumento da gordura corporal total e regional e a uma elevação da pressão arterial, predispondo assim às DCV.^7^

O tabagismo, o consumo excessivo de álcool e a adoção simultânea de 1 e ≥ 2 comportamentos de estilo de vida inadequados foram associados a maiores chances de presença de hipercolesterolemia. Evidências anteriores focaram na associação do consumo de álcool e tabagismo com marcadores lipídicos individuais, identificando interações desses fatores com níveis elevados de lipoproteína de baixa densidade (colesterol LDL).^33^ Considerando a coexistência frequente de tabagismo e consumo excessivo de álcool como comportamentos inadequados^11^ e sua capacidade individual de impactar negativamente os níveis lipídicos, sendo associados ao aumento dos triglicerídeos e às concentrações elevadas de colesterol LDL,^34^ é hipotetizado que os achados do presente estudo podem ser explicados pelos danos inerentes desses comportamentos inadequados.

A adoção de 1 comportamento de estilo de vida inadequado foi associada a maiores chances de presença de obesidade. Resultados análogos foram identificados na literatura, indicando que a adoção individual ou combinada de comportamentos de estilo de vida inadequados desempenha um papel substantivo no desenvolvimento da obesidade.^26^ A inatividade física e o consumo excessivo de álcool contribuem diretamente para um balanço energético positivo, resultando em aumento da adiposidade corporal.^26^ Além disso, hábitos alimentares inadequados levam à ingestão calórica excessiva que influencia os níveis séricos de marcadores inflamatórios, contribuindo assim para o acúmulo de tecido adiposo e o desenvolvimento da obesidade.^35^ Em relação ao tabagismo, o uso contínuo dessa substância pode estar associado a níveis elevados de cortisol, que estão relacionados ao acúmulo de gordura corporal.^26^ Além disso, o tabagismo pode estar associado ao aumento da resistência à insulina, estimulando a produção de hormônios hiperglicêmicos, que por sua vez estão associados ao aumento da adiposidade abdominal^36^ e ligados à obesidade.^26,36^

Em contraste com achados previamente relatados na literatura, que associaram a adoção de hábitos de vida saudáveis com uma redução na pressão arterial,^37,38^ o presente estudo identificou uma associação inversa nessa interrelação. Especificamente, a adesão simultânea a múltiplos hábitos de vida não saudáveis foi associada a uma menor probabilidade de hipertensão. Diversas hipóteses podem ser propostas para justificar esses resultados. Em primeiro lugar, especula-se que a associação inversa pode estar relacionada a determinantes dos níveis de pressão arterial não explorados no presente estudo, por exemplo, a influência de fatores genéticos que podem contribuir para que indivíduos não desenvolvam hipertensão, independentemente de fatores comportamentais.^39,40^ Segundo, considerando o caráter transversal do estudo e a ausência de informações sobre a duração que os participantes adotaram esses hábitos não saudáveis, especula-se que a associação inversa pode estar relacionada a um período insuficiente para que os hábitos não saudáveis impactassem negativamente a pressão arterial. O tempo necessário para os efeitos nocivos de hábitos não saudáveis impactarem os níveis de pressão arterial não é bem conhecido.^41^ Terceiro, considerando a natureza da pressão arterial, que é cronicamente impactada em conjunto com o aumento da idade,^42^ especula-se que a associação inversa entre hábitos de vida não saudáveis e menores chances de hipertensão pode estar relacionada à ampla faixa etária dos participantes do estudo, que incluiu tanto adultos jovens (menos propensos a alterações pressóricas) quanto idosos (mais propensos a alterações pressóricas). Quarto, a ampla disponibilidade de medicamentos gratuitos para pacientes diagnosticados com hipertensão, que contribui para o controle da pressão arterial elevada, é especulada como uma possível justificativa para a associação entre a adoção de hábitos não saudáveis e menores chances de hipertensão.^43^ No entanto, mudanças no estilo de vida, como a incorporação de hábitos saudáveis, são sugeridas para potencialmente eliminar a necessidade de medicamentos.^44^

O presente estudo apresenta pontos fortes que merecem ser destacados, por exemplo, a apresentação de dados abrangendo as cinco regiões geográficas do território brasileiro. Outro aspecto positivo considerado foi o desenho amostral utilizado (amostragem complexa), que atribuiu pesos diferentes à seleção probabilística dos elementos amostrais, resultando em estimativas mais precisas e representativas da população.^45^ Apesar dos pontos fortes, o presente estudo apresenta algumas limitações, entre elas o está o desenho transversal, que impede a determinação de causalidade e temporalidade nas associações avaliadas. O fato de as informações terem sido obtidas por meio de autorrelato constitui outra limitação. No entanto, as perguntas foram conduzidas por meio de instrumentos validados, o que confere qualidade às informações testadas.^22^

## Conclusão

A adoção de um maior número de comportamentos de estilo de vida inadequados foi associada a um aumento do número de fatores de risco para DCV. Tabagismo, consumo excessivo de álcool e a adoção simultânea de 1 e ≥ 2 comportamentos de estilo de vida inadequados foram associados a maiores chances para a presença de hipercolesterolemia. Adicionalmente, a adoção de 1 comportamento de estilo de vida inadequado foi associada a maiores chances para a presença de obesidade. Finalmente, a adesão simultânea a ≥ 2 comportamentos de estilo de vida não saudáveis foi associada a menores chances de presença de hipertensão. Esses achados sugerem uma interação complexa entre diferentes comportamentos de estilo de vida inadequados e seus impactos nos fatores de risco para DCV, reforçando a necessidade de abordagens multifacetadas na promoção de hábitos saudáveis para prevenir DCV.

## *Material suplementar

Para informação adicional, por favor, clique aqui.
