# Supplementary material for: Associação Individual e Simultânea entre Fatores de Risco para Doença Cardiovascular e Hábitos Inadequados do Estilo de Vida em uma Amostra do Brasil
Source: Arq Bras Cardiol. 2024 Oct 8;121(10):e20240149. [Article in Portuguese] doi: 10.36660/abc.20240149 (PMC11634217; doi:10.36660/abc.20240149)
Supplement: Supplementary file 1 [file 0066-782X-abc-121-10-e20240149-Suppl01.pdf]

Supplementary Material 1

Original article

Individual and joint association between cardiovascular disease risk factors and inadequate lifestyle behaviors in a sample from Brazil

Description of the sampling process:

For sampling purposes, the Municipal Human Development Index (mHDI) of all municipalities was identified, and subsequently used for classification: low mHDI (<0.550), medium mHDI (0.550-0.699), and high mHDI (>0.699) (BRASIL, 2019). From this classification, the social and economic development of each of the Brazilian municipalities with a presence of a Health Academy Program center was identified. With this information, it was possible to create 15 sampling strata. However, one of the created strata (low mHDI/Southern region of the country) did not have a municipality with a Health Academy Program center. Thus, 14 strata were established for the final sampling. According to the mHDI, three strata were created for the North, Northeast, Midwest, and Southeast regions, and two strata in the South region of the country.

The sampling process was conducted considering clusters in two stages. In the first stage, five municipalities within each stratum were randomly selected. The selected municipalities were contacted via the Brazilian Ministry of Health and municipal health departments. Thus, if the selected municipalities agreed to participate in the research, it was necessary to provide information about the number of users registered in the Health Academy Program. Based on this information, the second stage comprised the cluster sampling process, referring to the random selection of each user within the municipality that agreed to participate in the research. At this stage, the selected user was contacted by phone and invited to participate in the research. If the contacted user declined to participate in the research, there was a replacement of the user (new draw) within the stratum.

Based on the research budget, an estimated 86 interviews were planned for each sampling stratum, so that all strata had interviewed users. According to this information, the estimated sample was 1,204 users of the Health Academy Program in Brazil. In reality, 1,212 users were interviewed, as eight participants who had initially declined the invitation returned

the call to the research team informing that they would like to participate in the interview and were included in the final sample.
