# Supplementary material for: Associação Individual e Simultânea entre Fatores de Risco para Doença Cardiovascular e Hábitos Inadequados do Estilo de Vida em uma Amostra do Brasil
Source: Arq Bras Cardiol. 2024 Oct 8;121(10):e20240149. [Article in Portuguese] doi: 10.36660/abc.20240149 (PMC11634217; doi:10.36660/abc.20240149)
Supplement: Translation

# Translation: Individual and Joint Association between Cardiovascular Disease Risk Factors and Inadequate Lifestyle Behaviors in a Sample from Brazil

**DOI:** 10.36660/abc.20240149i

**Authors:** Letícia Gonçalves, Suellem Zanlorenci, Andreia Pelegrini, Tiago Rodrigues de Lima, Diego Augusto Santos Silva

**Affiliations:** 1 Florianópolis SC Brazil Universidade Federal de Santa Catarina, Florianópolis, SC – Brazil; 2 Florianópolis SC Brazil Universidade do Estado de Santa Catarina, Florianópolis, SC – Brazil

**Keywords:** Adult Health, Health of the Elderly, Health Risk Behaviors, Heart Disease Risk Factors

## Abstract

**Background:**

Cardiovascular diseases (CVD) are often influenced by modifiable factors, notably individuals’ lifestyle choices, which play a crucial role in modulating cardiovascular risk.

**Objective:**

To investigate the individual and simultaneous association between inadequate lifestyle behaviors and risk factors for CVD in adults and older adults.

**Method:**

A cross-sectional study with 1079 users of the Health Academy Program in Brazil. Information related to inadequate diet, excessive alcohol consumption, smoking, and physical inactivity were individually and collectively investigated (0, 1, or ≥ 2 factors) in association with CVD risk factors (hypertension, hypercholesterolemia, diabetes mellitus, and obesity), considering the following two outcomes: presence of CVD risk factors and number of CVD risk factors present in the same individual (0, 1, 2, or ≥ 3 risk factors). Logistic and multinomial logistic regression analyses were used. The statistical significance adopted was 5%.

**Results:**

A higher number of inadequate lifestyle behavior was associated with greater odds of simultaneous presence of 1, 2, or ≥ 3 CVD risk factors. The simultaneous adoption of 1 and ≥ 2 inadequate lifestyle behaviors was associated with greater odds of hypercholesterolemia. Simultaneous adherence to ≥ 2 inadequate lifestyle behaviors was associated with lower odds of hypertension.

**Conclusion:**

A greater number of inadequate lifestyle behaviors was associated with higher odds of simultaneous presence of multiple CVD risk factors.

## Introduction

Cardiovascular diseases (CVD) are a constant concern for public health, and they are among the leading causes of morbidity and mortality worldwide.^1,2^ These conditions are often influenced by modifiable factors, notably individuals’ lifestyle choices, which play a crucial role in modulating cardiovascular risk.^3,4^ Smoking,^5^ excessive alcohol consumption,^6^ physical inactivity,^3^ and inadequate dietary habits^7^ have been identified as the main elements responsible for the majority of deaths related to non-communicable diseases.^2,8^

Although the literature reveals an association between alcohol consumption and CVD, this relationship is still considered complex and, at times, paradoxical.^6^ On one hand, modest intake of alcoholic beverages has been associated with lower incidence and mortality from CVD, whereas excessive consumption of alcoholic beverages substantially increases the risk of CVD, on the other.^9^

Smoking is another variable that has been associated with an increased risk for outcomes directly related to CVD,^10,11^ including stroke, acute myocardial infarction, coronary heart disease, peripheral artery disease, heart failure, and hypertension.^11^ Smoking is also one of the main causes of chronic inflammatory state in the body, which contributes to the development of atherogenic disease processes and increases the levels of inflammatory biomarkers, known as indicators of cardiovascular events.^10^

Physical inactivity has been considered a well-established risk factor for the development of CVD, as highlighted by the United States Department of Health and Human Services in 2018.^12^ Regular physical activity not only has a positive impact on blood pressure levels,^13^ but also plays a crucial role in reducing the risk of obesity and diabetes.^14^

Dietary patterns have a significant impact on various risk factors related to cardiometabolic health, encompassing conditions such as heart diseases, stroke, and type 2 diabetes.^15^ These diseases collectively generate considerable impacts both on public health and economies.^16^ Understanding how dietary elements relate to cardiometabolic diseases is fundamental in establishing priorities, directing public health policies, and developing strategies that promote changes in eating habits, aiming to improve health.

Considering that unhealthy lifestyle habits can be modified^17^ and that individuals tend to simultaneously exhibit more than one unhealthy lifestyle habit,^18^ investigating such interrelationships contributes to the identification of subgroups of individuals who are more prone to CVD. This is particularly important as a higher number of unhealthy lifestyle habits is directly related to an increased risk of CVD.^11,19^

Therefore, the aim of the present study was to investigate the individual and joint association between inadequate lifestyle behaviors (inadequate diet, excessive alcohol consumption, smoking, and physical inactivity) with risk factors for CVD (hypertension, hypercholesterolemia, diabetes mellitus, and obesity) in a sample of adults and older adults from Brazil.

## Method

### Study design, ethical considerations, and participants

The present cross-sectional study utilized data from the population study named "MOTIVA-SUS: a cross-sectional epidemiological study on the motivational determinants for physical activity among users of the Health Academy Program." The study was approved by the Human Research Ethics Committee at Universidade Federal de Santa Catarina. Data collection was conducted between February and August 2022, through telephone interviews, due to the COVID-19 pandemic situation. All research participants received information contained in the free and informed consent form, and only those who agreed with the information participated in the interview. The study included a representative sample of individuals aged 18 years or older, of both sexes, users of the Health Academy Program from the five geographical regions of Brazilian territory (Central-West, Northeast, North, Southeast, and South). The description of the sampling process is detailed in Supplementary Material 1.

The following exclusion criteria were considered: bedridden subjects, amputees, those in casts, and individuals who did not have the cognitive capacity to communicate via telephone contact. Refusals or losses were considered for eligible individuals who could not be reached after a minimum of four calls, one of which was on the weekend and another in the evening. Additionally, refusals were considered when eligible individuals declined to participate in the study, even after clarification about the purpose of the research.

### Cardiovascular disease risk factors

Body mass index (BMI) was initially noted as a continuous variable and later dichotomized. Individuals self-reported body mass and height. From this information, BMI (kg/m^2^) was calculated. Obesity was classified as a BMI ≥ 30.0 kg/m^2^. ^20^

Information obtained in the interviews was used to identify individuals with hypertension, dyslipidemia, and diabetes mellitus, based on affirmative responses to the following question regarding self-reported medical diagnosis: "Has a doctor or healthcare professional ever told you that you have…?" Additionally, the use of specific medication for each of these conditions was used to classify the subjects as positive for the investigated risk factors.

### Inadequate lifestyle behaviors

Variables related to the adoption of inadequate lifestyle behaviors (smoking, excessive alcohol consumption, physical inactivity, and inadequate diet) were collected based on questions derived from the Brazilian Surveillance of Risk and Protective Factors for Chronic Diseases by Telephone Survey (VIGITEL) questionnaire.^21^

Smoking was assessed using the following question: "Do you currently smoke?" with response options of "no" or "yes." Individuals who responded "yes" were considered smokers, regardless of the intensity or frequency of the smoking habit.^21^

Excessive alcohol consumption was defined as the intake of 4 or more alcoholic beverage doses on a single occasion, at least once in the last 30 days, as assessed by the affirmative response to the following question: "In the last 30 days, have you consumed 4 or more doses of alcoholic beverages on a single occasion?" Although the literature does not present a defined consensus on alcohol consumption recommendations for adults,^20^ a metric commonly adopted to characterize excessive consumption is the intake of 4 or more doses of alcoholic beverages on a single occasion, occurring at least once in the last 30 days.^20^

Physical inactivity was assessed based on the leisure domain, using the following question: "In the last 3 months, have you practiced any type of physical exercise or sport?" The response options were "no" or "yes." According to recommendations in the literature, individuals were classified as "physically inactive" if they had not practiced or performed any physical activity during leisure time in the last 3 months.^21,22^ The reproducibility of VIGITEL questions related to physical activity showed a specificity of 72.0% to 91.2% and a sensitivity of 54.8% to 67.7% for inactive individuals, demonstrating reliability in this domain.^22^

Variables associated with dietary habits encompassed the frequency of food consumption on the day prior to the interview, which were limited, for the purposes of this study, to groups of industrialized/ultra-processed foods,^21,23^ obtained from the following questions: "Now I am going to list some foods and I would like you to tell me if you ate any of them yesterday (from when you woke up to when you went to sleep): soda; fruit juice from a carton, box or can; powdered drink mixes; chocolate drinks; flavored yogurt; packaged snacks (or chips) or salty biscuits/crackers; biscuit/sweet cookies, stuffed cookies, or packaged cakes; chocolate, ice cream, gelatin, or other industrialized desserts; sausage, salami, mortadella, or ham; sliced bread, hot dog bread, or hamburger bread; mayonnaise, ketchup, or mustard; margarine; instant noodles, packaged soup, frozen lasagna, or other ready-made dish purchased frozen." The answer options were "yes" or "no." Those who consumed 5 or more groups of ultra-processed foods on the day prior to the interview were considered as having an "inadequate diet."^21^

Information regarding inadequate lifestyle behaviors was investigated both individually and collectively (according to the number of inadequate behaviors). For this purpose, the number of inadequate lifestyle behaviors adopted by each subject (inadequate diet, excessive alcohol consumption, smoking, and physical inactivity) was summed up and transformed into an ordinal scale (0 = no inadequate lifestyle behavior; 1 = 1 inadequate lifestyle behavior; 2 = 2 inadequate lifestyle behaviors; 3 = 3 inadequate lifestyle behaviors). However, since a small number of individuals adopted 3 inadequate lifestyle behaviors simultaneously (n = 12; 1.1%), for analysis purposes, the categories 2 and 3 were grouped together (0, 1, or ≥ 2 inadequate lifestyle behaviors).

### Dependent variables

Two different outcomes were considered for this study: risk factors for CVD (hypertension, dyslipidemia, diabetes mellitus, and obesity) and the number of CVD risk factors present in the same individual (0, 1, 2, 3, or 4 risk factors). As less than 3.0% (n = 31) of the sample had 4 CVD risk factors, they were grouped into the category of 3 CVD risk factors. Thus, for analysis purposes, subjects in this study were classified as having 0, 1, 2, or ≥ 3 CVD risk factors.

### Sociodemographic and cardiovascular health variables

The control variables analyzed included sex (male/female), age group (adults, defined as 20 to 59 years of age; older adults, defined as 60 years or older), education (0 to 8 years, 9 to 11 years, 12 years or more), and marital status (single, married/domestic partnership, separated/divorced/widowed). Based on self-reported medical diagnosis, adults and older adults were classified as positive for CVD when they affirmed having a history of stroke, cerebral ischemia, or heart disease.

### Data analysis

Descriptive statistics were used to describe the analyzed information. Categorical variables are described through absolute and relative frequencies. The chi-square test of heterogeneity was used to identify possible differences between groups according to the individual presence and the number of CVD risk factors present in the same individual.

Binary logistic regression was used to test the association between the presence of CVD risk factors (hypertension, hypercholesterolemia, diabetes mellitus, and obesity) as dependent variables with individual adoption and simultaneous (0, 1, ≥ 2 habits) adoption of inadequate lifestyle habits (smoking, physical inactivity, inadequate diet, and excessive alcohol consumption) by the same individual as independent variables. For this regression model, the results were expressed as odds ratio (OR) with a 95% confidence interval (CI), with the reference category being absence of the disease.

Additionally, multinomial logistic regression analysis was employed to investigate the association between the simultaneous presence of CVD risk factors in the same individual (dependent variable) with individual adoption and simultaneous adoption of inadequate lifestyle behaviors (independent variables), with results expressed as OR and 95% CI, with the reference category being 0 CVD risk factors.

The association analyses were adjusted considering sex, age group, years of education, marital status, and CVD (cardiovascular disease, cerebral ischemia, and stroke), regardless of the statistical significance level of the crude analysis in association with the outcomes.

The analyses were performed considering the sample weights and the study design. Data analysis was conducted using Stata software (Stata Corp LP, College Station, TX, USA), version 16.0, with a p value of < 0.05 being considered statistically significant.

## Results

The study included 1079 individuals with complete information for all investigated variables (89.0% of the original sample). Of the total evaluated, 40.4% were hypertensive; 36.6% had hypercholesterolemia; 20.0% had diabetes mellitus; and 32.9% were obese. In addition, 28.4% of the sample did not present any CVD risk factor, while 31.4% had 1 CVD risk factor; 24.9% had 2 CVD risk factors; and 15.3% had 3 or more CVD risk factors simultaneously (Table 1). Table 2 presents the distribution of the sample for each of the investigated CVD risk factors.

**Table 1:** Descriptive information regarding participants in the Health Academy Program. Brazil, 2022

Variables		n	% (95% CI)
**Sex**			
	Male	81	6.8 (2.8-15.7)
	Female	998	93.2 (84.3-97.2)
**Age (years)**			
	20-59	804	74.4 (55.1-87.3)
	≥ 60	275	25.6 (12.6-44.9)
**Years of study**			
	0-8	204	18.9 (11.7-28.4)
	9-11	216	20.0 (18.9-22.4)
	≥ 12	659	91.1 (50.7-69.8)
**Marital status**			
	Single	223	20.1 (10.6-34.5)
	Married	668	62.9 (59.3-66.4)
	Separate/widow/widower	188	17.0 (9.7-28.0)
**Smoking**			
	No	1052	97.6 (97.1-97.9)
	Yes	27	2.4 (2.0-2.8)
**Inadequate diet**			
	No	931	85.3 (80.1-89.3)
	Yes	148	14.6 (10.6-19.8)
**Physical inactivity**			
	No	750	69.9 (57.8-79.7)
	Yes	329	30.1 (20.3-42.2)
**Excessive alcohol consumption**			
	No	908	84.3 (82.2-86.2)
	Yes	171	15.6 (13.7-17.7)
**Simultaneous adoption of inadequate lifestyle habits**			
	0	327	29.5 (24.3-35.2)
	1	634	59.3 (57.5-61.0)
	≥ 2	118	11.2 (7.9-15.6)
**Arterial hypertension**			
	No	655	59.6 (49.6-68.8)
	Yes	424	40.4 (31.2-50.4)
**Hypercholesterolemia**			
	No	689	63.4 (57.5-69.9)
	Yes	390	36.6 (31.1-42.5)
**Diabetes mellitus**			
	No	877	80.0 (76.0-83.4)
	Yes	202	20.0 (16.6-24.0)
Obesity*			
	No	735	67.1 (58.5-74.6)
	Yes	344	32.9 (25.4-41.4)
**Simultaneous presence of risk factors for CVD**			
	0	324	28.4 (23.3-34.1)
	1	340	31.4 (30.2-32.6)
	2	256	24.9 (18.7-32.3)
	≥ 3	159	15.3 (14.0-16.6)
**Presence of CVD**			
	No	987	91.8 (83.7-96.0)
	Yes	92	8.2 (3.9-16.2)

* information generated from body mass index (≥ 30 kg/m^2^).

CI: confidence interval; n: number of frequencies; presence of CVD: cardiovascular disease, stroke, or cerebral ischemia.

**Table 2:** Distribution and frequency of inadequate lifestyle habits analyzed individually or as a group of factors, n = 1079

Inadequate lifestyle habits	n	(%)
None	534	49.5
Smoking	9	0.8
Inadequate diet	73	6.8
Physical inactivity	234	21.6
Excessive alcohol consumption	111	10.3
Smoking + inadequate diet	3	0.3
Smoking + physical inactivity	8	0.7
Smoking + excessive alcohol consumption	2	0.2
Inadequate diet + physical inactivity	45	4.2
Inadequate diet + excessive alcohol consumption	18	1.7
Physical inactivity + excessive alcohol consumption	30	2.8
Smoking + inadequate diet + physical inactivity	2	0.2
Smoking + inadequate diet + excessive alcohol consumption	-	-
Smoking + physical inactivity + excessive alcohol consumption	3	0.3
Inadequate diet + physical inactivity + excessive alcohol consumption	7	0.6
Smoking + inadequate diet + physical inactivity + excessive alcohol consumption	-	-

The presence of 3 or more CVD risk factors was higher among participants who were female, were 20 to 59 years old, had 12 or more years of education, were married, adopted 1 inadequate lifestyle behavior, and did not report presence of CVD (Table 3 and Table 4).

**Table 3:** Descriptive information on individual characteristics and lifestyle habits according to risk factors for cardiovascular diseases in participants of the Health Academy Program. Brazil, 2022

Variables		Hypertension		p value	Hypercholesterole		p value	Diabetes mellitus		p value	Obesity*		p value
		n	% (95% CI)		n	% (95% CI)		n	% (95% CI)		n	% (95% CI)	
**Sex**				<0.01†			0.99			0.51			0.13
	Male	35	8.4 (3.9-17.2)		27	6.8 (2.9-15.1)		18	8.9 (3.0-23.4)		21	5.6 (1.7-16.7)	
	Female	389	91.6 (82.8-96.1)		363	93.2 (84.9-97.1)		184	91.1 (76.6-97.0)		323	94.4 (83.3-98.3)	
**Age (years)**				<0.01†			<0.01†			<0.01†			<0.01†
	20-59	254	60.5 (45.1-74.1)		259	67.1 (50.0-80.5)		118	59.5 (41.6-75.2)		280	84.1 (61.8-94.5)	
	≥ 60	170	39.5 (25.8-54.9)		131	32.9 (19.5-49.9)		84	40.5 (24.7-58.4)		64	15.9 (5.5-38.2)	
**Years of study**				<0.01†			0.08			<0.01†			0.84
	0-8	112	26.9 (18.4-37.6)		89	22.9 (14.5-34.2)		47	22.9 (16.7-30.6)		69	19.3 (10.5-32.6)	
	9-11	101	24.3 (20.9-27.9)		88	22.2 (17.5-27.7)		57	28.4 (26.4-30.4)		65	20.5 (16.9-24.6)	
	≥ 12	211	48.8 (38.3-59.3)		213	54.9 (49.9-59.7)		98	48.7 (41.3-56.2)		210	60.2 (51.7-68.1)	
**Marital status**				<0.01†			<0.01†			0.13			<0.01†
	Single	66	15.8 (9.3-25.6)		55	13.7 (6.7-25.8)		34	18.7 (9.8-32.7)		76	23.5 (14.1-36.4)	
	Married	266	63.9 (62.0-65.7)		255	66.0 (65.2-66.8)		129	65.0 (62.3-67.6)		216	63.1 (60.9-65.2)	
	Separate/widow/widower	92	20.2 (13.1-29.9)		80	20.3 (12.3-31.7)		39	16.3 (8.6-28.7)		52	13.4 (6.4-25.9)	
**Smoking**				0.21			0.06			0.15			0.96
	No	407	96.1 (92.1-98.1)		373	95.9 (93.3-97.4)		193	95.5 (90.1-98.0)		334	97.6 (96.7-98.3)	
	Yes	17	3.9 (1.9-7.9)		17	4.1 (2.5-6.6)		9	4.5 (2.0-9.8)		10	2.4 (1.7-3.3)	
**Inadequate diet**				0.11			0.06			0.29			0.60
	No	373	88.4 (78.0-94.2)		335	84.5 (78.8-88.9)		176	88.0 (76.3-94.3)		294	84.4 (74.3-91.0)	
	Yes	51	11.6 (5.8-21.9)		55	15.5 (11.0-21.2)		26	12.0 (5.7-23.6)		50	15.6 (8.9-25.6)	
**Physical inactivity**				0.79			0.40			0.06			0.96
	No	298	70.2 (56.5-81.1)		278	71.7 (58.3-82.2)		151	76.4 (58.5-88.2)		241	69.9 (60.2-78.1)	
	Yes	126	29.8 (18.9-43.5)		112	28.3 (17.8-41.7)		51	23.6 (11.8-41.4)		103	30.1 (21.9-39.8)	
**Excessive alcohol consumption**				0.88			0.03†			0.27			0.63
	No	335	84.2 (79.5-87.9)		322	82.2 (78.7-85.1)		175	86.2 (81.3-89.9)		289	83.4 (81.2-85.4)	
	Yes	69	15.8 (12.1-20.5)		68	17.8 (14.8-21.2)		27	13.8 (10.0-18.7)		55	16.6 (14.6-18.8)	
**Simultaneous adoption of inadequate lifestyle habits**				<0.01†			<0.01†			0.02†			0.01†
	0	0	-		91	23.8 (17.5-31.5)		43	23.2 (16.4-31.8)		87	23.7 (16.7-32.3)	
	1	384	90.7 (82.0-95.5)		251	63.4 (59.7-66.9)		138	66.5 (60.6-71.9)		218	64.5 (59.5-69.2)	
	≥ 2	40	9.3 (4.5-18.0)		48	12.8 (9.3-17.3)		21	10.3 (6.4-16.2)		39	11.8 (8.9-15.4)	
**Presence of CVD**				<0.01†			0.09			0.02†			0.31
	No	363	86.1 (74.6-92.9)		345	89.5 (77.3-95.5)		174	86.9 (73.5-94.1)		309	90.4 (76.7-96.3)	
	Yes	61	13.9 (7.1-25.3)		45	10.5 (4.4-22.6)		28	13.1 (5.9-26.4)		35	9.6 (3.6-23.2)	

Chi-square test of heterogeneity. CI: confidence interval; n: number of frequencies; presence of CVD: cardiovascular disease, stroke, and cerebral ischemia.

* information generated from body mass index (obesity ≥ 30 kg/m^2^);

† p < 0.05.

**Table 4:** Descriptive information on individual characteristics and lifestyle habits according to the simultaneous presence of risk factors for cardiovascular diseases in participants of the Health Academy Program. Brazil, 2022

Variables		Simultaneous presence of risk factors for CVD								p value
		0		1		2		≥ 3		
		n	% (95% CI)	n	% (95% CI)	n	% (95% CI)	n	% (95% CI)	
**Sex**										0,04†
	Male	32	8,5 (2,9-21,8)	17	4,0 (1,8-8,5)	16	6,5 (2,0-18,7)	16	10,3 (3,7-25,2)	
	Female	292	91,5 (78,2-97,0)	323	96,0 (91,4-98,2)	240	93,5 (81,3-97,9)	143	89,7 (74,8-96,5)	
**Age (years)**										<0,01†
	20-59	279	86,6 (72,6-94,0)	256	73,9 (47,5-89,9)	169	65,5 (51,0-77,6)	100	67,4 (42,6-85,2)	
	≥ 60	45	13,4 (5,9-27,4)	54	26,1 (10,1-52,5)	87	34,5 (22,4-49,0)	59	32,5 (14,8-57,3)	
**Years of study**										<0,01†
	0-8	44	13,4 (8,3-20,8)	59	16,0 (10,0-24,5)	55	21,9 (13,2-34,0)	46	28,8 (19,0-41,0)	
	9-11	44	13,6 (12,0-15,4)	77	23,7 (19,7-28,1)	56	22,3 (19,9-24,8)	39	24,7 (18,6-31,9)	
	≥ 12	236	72,9 (65,2-79,5)	204	60,3 (49,0-70,6)	145	55,8 (44,5-66,5)	74	46,5 (39,9-53,2)	
**Marital status**										0,03†
	Single	93	27,4 (14,9-44,9)	60	16,7 (9,3-28,2)	44	16,2 (5,9-36,9)	26	19,4 (12,8-28,3	
	Married	186	58,3 (47,3-68,5)	223	66,2 (62,7-69,5)	153	62,0 (54,4-69,0)	106	66,3 (64,8-67,8)	
	Separate/widow/widower	45	14,2 (8,9-22,0)	57	17,1 (8,9-29,9)	59	21,8 (12,5-35,2)	27	14,2 (7,9-24,3)	
**Smoking**										0,14
	No	320	99,1 (94,7-99,8)	333	97,7 (93,3-99,3)	251	98,1 (93,9-99,4)	148	93,7 (87,6-96,9)	
	Yes	4	0,9 (0,1-5,3)	7	2,3 (0,7-6,7)	5	1,9 (1,0-6,1)	11	6,3 (3,1-12,3)	
**Inadequate diet**										0,38
	No	276	83,8 (80,7-86,4)	293	84,4 (80,0-87,9)	227	88,2 (79,7-93,4)	135	85,5 (70,0-93,7)	
	Yes	48	16,2 (13,5-19,3)	47	15,6 (12,0-19,9)	29	11,8 (6,6-20,3)	24	14,5 (6,3-29,9)	
**Physical inactivity**										0,23
	No	224	69,5 (56,9-79,7)	228	67,5 (56,4-76,9)	181	70,5 (53,2-83,4)	117	74,5 (63,0-83,3)	
	Yes	100	30,5 (20,3-43,1)	112	32,5 (23,1-43,6)	75	29,5 (16,5-46,8)	42	25,5 (16,6-37,0)	
**Excessive alcohol consumption**										0,24
	No	265	83,2 (73,9-89,6)	303	88,5 (82,9-92,4)	209	81,3 (79,9-82,5)	131	83,0 (75,3-88,6)	
	Yes	59	16,8 (10,3-26,0)	37	11,5 (7,6-17,1)	47	18,7 (17,5-20,1)	28	17,0 (11,3-24,7)	
**Simultaneous adoption of inadequate lifestyle habits**										<0,01†
	0	154	47,2 (40,6-53,9)	131	37,3 (30,4-44,7)	36	14,4 (7,4-26,2)	6	4,9 (1,0-20,4)	
	1	132	42,0 (35,8-48,3)	176	51,5 (47,6-55,4)	193	73,3 (69,0-77,3)	133	84,7 (80,7-87,9)	
	≥ 2	38	10,8 (7,9-14,6)	33	11,2 (8,0-15,4)	27	12,2 (7,3-19,6)	20	10,3 (5,7-18,1)	
**Presence of CVD**										<0,01†
	No	307	94,5 (88,2-97,5)	324	96,1 (92,6-98,0)	224	88,8 (81,7-93,4)	132	82,9 (59,5-94,1)	
	Yes	17	5,5 (2,5-11,8)	16	3,9 (1,9-7,4)	32	11,2 (6,6-18,3)	27	17,1 (5,8-40,5)	

Chi-square test of heterogeneity; n: number of frequencies; %: percentage; ≥: greater than or equal; Presence of CVD: Cardiovascular Disease. Stroke. Stroke or Cerebral Ischemia; 95%CI: confidence interval;

† p<0.05.

Smoking, excessive alcohol consumption, and the adoption of 1 and ≥ 2 inadequate lifestyle behaviors were associated with higher odds of hypercholesterolemia. The adoption of 1 inadequate lifestyle behavior was associated with higher odds of obesity. The simultaneous adoption of ≥ 2 inadequate lifestyle behaviors was associated with lower odds of hypertension (Table 5).

**Table 5:** Adjusted binary logistic regression analysisa between the adoption of inadequate lifestyle habits and the presence of risk factors for cardiovascular disease in adults and elderly users of the Health Academy Program. Brazil, 2022

Variables		Hypertension^b^		Hypercholesterole^b^		Diabetes mellitus^b^		Obesity* b	
		OR	% (95% CI)	OR	% (95% CI)	OR	% (95% CI)	OR	% (95% CI)
**Smoking**^a^									
	No	1		1		1		1	
	Yes	1.1	0.2-4.9	2.0	1.1-3.6†	2.3	0.9-5.8	0.7	0.4-1.5
**Physical inactivity**^a^									
	No	1		1		1		1	
	Yes	1.1	0.8-1.4	0.9	0.6-1.3	0.7	0.4-1.1	0.9	0.7-1.2
**Inadequate diet**^a^									
	No	1		1		1		1	
	Yes	0.6	0.4-1.2	1.1	1.0-1.3	0.7	0.4-1.3	1.1	0.7-1.8
**Excessive alcohol consumption**^a^									
	No	1		1		1		1	
	Yes	1.2	0.9-1.4	1.4	1.2-1.6†	0.9	0.6-1.3	1.0	0.7-1.7
**Simultaneous adoption of inadequate lifestyle habits**^a^									
	0	‡		1		1		1	
	1	1		1.4	1.1-1.9†	1.3	0.9-1.9	1.7	1.2-2.4†
	≥2	0.3	0.2-0.6†	1.7	1.4-2.1†	1.1	0.9-1.4	1.5	0.9-2.6

CI: confidence interval; OR: odds ratio.

* information generated from body mass index (obesity ≥ 30 kg/m^2^);

† p < 0.05;

‡ absence of individuals who simultaneously had hypertension and did not adopt any inadequate lifestyle habits. For this situation, the category of individuals who adopted 1 inadequate lifestyle habit was used as a reference.

a Results adjusted for sex, age, schooling, marital status, and cardiovascular disease;

b Absence of the disease was considered a reference for the analyses.

Higher odds for the presence of 1 CVD risk factor were observed among those who adopted 1 and ≥ 2 inadequate lifestyle behaviors. Regarding the findings related to the presence of 2 CVD risk factors, higher odds for this condition were observed among those who had 1 and ≥ 2 inadequate lifestyle behaviors. Higher odds for the presence of 3 CVD risk factors were observed among those who adopted 1 and ≥ 2 inadequate lifestyle behaviors (Table 6).

**Table 6:** Adjusted multinominal logistic regression analysisa between the adoption of inadequate lifestyle habits and the presence of risk factors for cardiovascular disease in adults and elderly users of the Health Academy Program. Brazil, 2022

Variables		Simultaneous adoption of inadequate lifestyle habits					
		1^b^		2^b^		≥ 3^b^	
		OR	% (95% CI)	OR	% (95% CI)	OR	% (95% CI)
**Smoking**^a^							
	No	1		1		1	
	Yes	2.1	0.6-7.1	1.7	0.3-11.9	5.8	0.5-59.4
**Physical inactivity**^a^							
	No	1		1		1	
	Yes	1.2	0.9-1.5	1.0	0.7-1.6	0.8	0.5-1.3
**Inadequate diet**^a^							
	No	1		1		1	
	Yes	1.0	0.8-1.3	0.7	0.4-1.2	0.9	0.4-2.1
**Excessive alcohol consumption**^a^							
	No	1		1		1	
	Yes	0.7	0.3-1.8	1.3	0.7-2.3	1.2	1.0-1.4
**Simultaneous adoption of inadequate lifestyle habits**^a^							
	0	1		1		1	
	1	1.5	1.1-2.0†	5.3	3.3-8.5†	18.0	3.4-94.6†
	≥2	1.4	1.1-1.9†	4.0	1.9-8.3†	9.6	1.5-62.6†

CI: confidence interval; CVD: cardiovascular disease; OR: odds ratio.

† p < 0.05.

a Results adjusted for sex, age, schooling, marital status, and cardiovascular disease;

b Presence of 0 risk factors for CVD was considered a reference for the analyses.

## Discussion

The primary finding of this study was that a greater number of inadequate lifestyle behaviors were associated with higher odds for the simultaneous presence of 1, 2, or ≥ 3 CVD risk factors. The potential mechanisms related to the harmful effects on cardiovascular health derived from the adoption of inadequate lifestyle behaviors may be related to the combination of health detriments attributed to each factor. Physical inactivity directly contributes to positive energy balance, which is associated with worsened lipid and lipoprotein profiles^3,24,25^ and increased body adiposity,^26^ as well as insulin sensitivity and imbalances in blood pressure levels.^27^ Excessive alcohol consumption can contribute to an increase in systolic blood pressure through increased arterial stiffness^28^ and contribute to metabolic alterations that can in turn directly increase the risk for CVD.^29^ The deleterious effects of smoking on cardiovascular health may be related to the chemical components present in tobacco, such as nicotine, tar, and carbon monoxide.^30^ These components, in addition to being directly associated with sympathetic nervous activation and increased blood pressure,^31^ intensify insulin resistance, establishing a relationship with central fat accumulation, which contributes to the development of metabolic disorders such as metabolic syndrome and diabetes mellitus.^30^ Additionally, the adoption of an inadequate diet^32^ can lead to imbalances in glycemic metabolism, increased total and regional body fat, and elevated blood pressure, predisposing to CVD.^7^

Smoking, excessive alcohol consumption, and the simultaneous adoption of 1 and ≥ 2 inadequate lifestyle behaviors were associated with higher odds for the presence of hypercholesterolemia. Previous evidence focused on the association of alcohol consumption and smoking with individual lipid markers, identifying interactions of these factors with elevated levels of low-density lipoprotein (LDL cholesterol).^33^ Considering the frequent coexistence of smoking and excessive alcohol consumption as inadequate behaviors^11^ and their individual capacity to negatively impact lipid levels, with smoking and excessive alcohol consumption associated with increases in triglycerides and elevated LDL-cholesterol concentrations,^34^ it is hypothesized that the findings of the present study can be explained by the inherent harm of these inadequate behaviors.

The adoption of 1 inadequate lifestyle behavior was associated with higher odds for the presence of obesity. Analogous results have been identified in the literature, indicating that the individual or combined adoption of inadequate lifestyle behaviors plays a substantive role in the development of obesity.^26^ Physical inactivity and excessive alcohol consumption directly contribute to a positive energy balance, resulting in increased body adiposity.^26^ Additionally, inadequate dietary habits lead to excessive caloric intake that influences serum levels of inflammatory markers, thus contributing to the accumulation of adipose tissue and the development of obesity.^35^ Regarding smoking, continuous use of this substance may be associated with elevated cortisol levels, which are related to the accumulation of body fat.^26^ Furthermore, smoking may be associated with increased insulin resistance, stimulating the production of hyperglycemic hormones, which are in turn associated with increased abdominal adiposity^36^ and linked to obesity.^26,36^

In contrast to previously reported findings in the literature, which associated the adoption of healthy lifestyle habits with a reduction in blood pressure,^37,38^ the present study identified an inverse association in this interrelationship. Specifically, the simultaneous adherence to multiple unhealthy lifestyle habits was associated with a lower likelihood of hypertension. Several hypotheses can be proposed to justify these results. First, it is speculated that the inverse association may be related to determinants of blood pressure levels not explored in the present study, such as the influence of genetic factors that can contribute to individuals not developing hypertension regardless of behavioral factors.^39,40^ Second, considering the cross-sectional nature of the study and the absence of information regarding the duration that the participants had adopted these unhealthy habits, it is speculated that the inverse association may be related to an insufficient period for the unhealthy habits to negatively impact blood pressure. The required time for the harmful effects of unhealthy habits to impact blood pressure levels is not well known.^41^ Third, considering the nature of blood pressure, which is chronically impacted in conjunction with increasing age,^42^ it is speculated that the inverse association between unhealthy lifestyle habits and lower chances of hypertension may be related to the broad age range of the study participants, which included both young adults (less prone to blood pressure changes) and the elderly (more prone to blood pressure changes). Fourth, the widespread availability of free medications for patients diagnosed with hypertension, which contributes to the control of elevated blood pressure, is speculated as a possible justification for the association between the adoption of unhealthy habits and lower chances of hypertension.^43^ However, lifestyle changes, such as the incorporation of healthy habits, are suggested to potentially eliminate the need for medication.^44^

This study presents strengths that should be highlighted, such as the presentation of data covering the five geographical regions of the Brazilian territory. Another positive aspect considered was the sample design used (complex sampling), which attributed different weights to the probabilistic selection of sample elements, resulting in more precise and representative estimates of the population.^45^ Despite the strengths, this study has some limitations, which include the cross-sectional design, which prevents the determination of causality and temporality in the associations evaluated. The fact that information was obtained through self-report is another limitation. However, the questions were conducted using validated instruments, which confers quality to the tested information.^22^

## Conclusion

The adoption of a greater number of inadequate lifestyle behaviors was associated with an increase in the number of CVD risk factors. Smoking, excessive alcohol consumption, and the simultaneous adoption of 1 and ≥ 2 inadequate lifestyle behaviors were associated with higher odds for the presence of hypercholesterolemia. Additionally, the adoption of 1 inadequate lifestyle behavior was associated with higher odds for the presence of obesity. Finally, the simultaneous adherence to ≥ 2 unhealthy lifestyle behaviors was associated with lower odds for the presence of hypertension. These findings suggest a complex interaction between different inadequate lifestyle behaviors and their impacts on CVD risk factors, reinforcing the need for multifaceted approaches in promoting healthy habits to prevent CVD.

## *Supplemental Material

For additional information, please click here.
